# First person – Jack Barrington

**DOI:** 10.1242/dmm.052638

**Published:** 2025-09-30

## Abstract

First Person is a series of interviews with the first authors of a selection of papers published in Disease Models & Mechanisms, helping researchers promote themselves alongside their papers. Jack Barrington is first author on ‘ Interleukin-1 regulates myeloid cell trafficking and cerebral blood flow following intracerebral haemorrhage’, published in DMM. Jack conducted the research described in this article while a PhD student in Stuart M. Allan's lab at The University of Manchester, Manchester, UK. He is now a postdoctoral research fellow in the lab of Barry McColl at The University of Edinburgh, Edinburgh, UK, investigating immune contributions to brain health and disease.


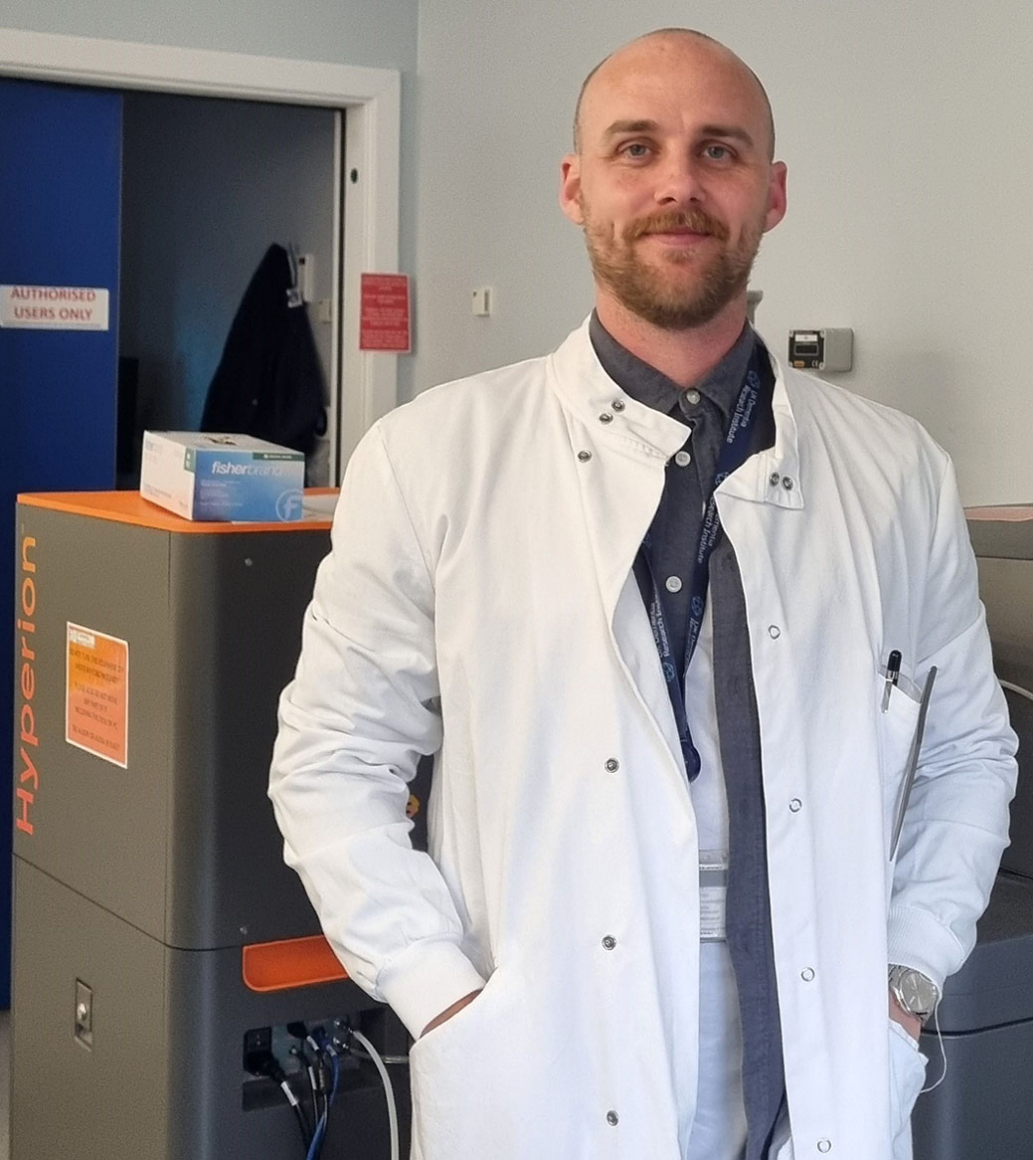


**Jack Barrington**

**Who or what inspired you to become a scientist?**

I was gifted a microscope by my grandfather for my seventh birthday, and observing the hidden natural beauty of biology with it is one of my earliest scientific memories! I have always enjoyed problem solving, and my family/teachers really encouraged me to pursue those interests in both science and maths. I think it was the enthusiasm of my high school and college biology tutors that pulled me to science over other subjects at those life stages. Since then, I have been very fortunate to meet and be mentored by amazing people along my scientific journey. I was encouraged to pursue a PhD by Professors John Aplin and Christoph Ballestrem at The University of Manchester, both providing me with the confidence that I could be a researcher. I then landed in one of the best places in Manchester's Brain Inflammation Group to learn to become a scientist during my PhD, and I have not looked back since then!

**What is the main question or challenge in disease biology you are addressing in this paper? How did you go about investigating your question or challenge?**

Intracerebral haemorrhage (ICH) is the second leading cause of stroke and occurs when a diseased cerebral blood vessel bursts and blood enters the brain parenchyma. It contributes a disproportionate amount of death and disability due to stroke worldwide. One reason for this is we have no specific medical interventions for ICH, despite several life-saving therapies being available for the leading cause of stroke, ischaemia. There are many studies showing that recruitment of circulating myeloid cells following ICH contributes to brain damage during acute phases. However, we currently lack a therapeutic target to prevent brain recruitment of all myeloid cell subtypes. In this study, we aimed to deliver a therapeutic target by first identifying and subsequently inhibiting the main molecular pathway(s) promoting myeloid cell recruitment during acute phases. To do this, omics approaches and immunostaining were combined in mouse and human brain tissue, revealing interleukin-1 (IL-1) as a potential upstream regulator of acute myeloid cell recruitment. IL-1 was blocked using the naturally occurring IL-1 receptor antagonist in a mouse model, resulting in fewer myeloid cells reaching the brain and reduced cerebral blood flow at 24 h, along with worse neuromotor outcomes up to 7 days. Our results really highlight the need for more studies on ICH and specifically around the brain immune–vascular interactions. I believe that the immune and coagulation systems have co-evolved important beneficial interactions, but there would be very little pressure for these interactions to work as well in brain tissue, so we have a lot of work to do to delineate all of that!

We used our science detective skills to trace the connections between the molecules that change after haemorrhage and found one main suspect called IL-1 that connected the most dots

**How would you explain the main findings of your paper to non-scientific family and friends?**

There are two main types of stroke. One is caused by the brain's blood vessels becoming clogged, known as ischaemic stroke; the other is the result of the brain's blood vessel bursting and is known as haemorrhagic stroke. There are no treatments for haemorrhagic stroke, but there is some evidence that blocking the immune system might improve the disease in experimental models. We tried to find the key molecule that signals to the immune system to enter the brain after haemorrhage, believing if we can block this molecule then we might be able to block the bad actions of the immune system. We used a tool that allows us to see how all the molecules in the brain change after haemorrhage in a mouse model. We used our science detective skills to trace the connections between the molecules that change after haemorrhage and found one main suspect called IL-1 that connected the most dots. We showed that IL-1 was there in human brain tissue following haemorrhage, and blocking it in our mouse model resulted in fewer immune cells getting into the brain. To our surprise, mice fared worse when we blocked IL-1. We are not too sure why this is the case, but we think IL-1 might also control how much blood flows to the brain. The immune system clearly has some complicated actions following brain haemorrhage, so we need to do more science to understand it better!

**What are the potential implications of these results for disease biology and the possible impact on patients?**

We emphasise caution when interpreting our results and comparing with the human condition. In our intervention phase, we blocked IL-1 from the onset of ICH, and this is not how a treatment would be given in the clinical setting. A delayed treatment regimen is essential to better assess the translatability of an IL-1-blocking therapy in ICH. Our data do add to growing evidence the inflammatory response has some beneficial functions during acute ICH, and these interactions will need further study to fully tease apart beneficial from damaging functions.

**Figure:**
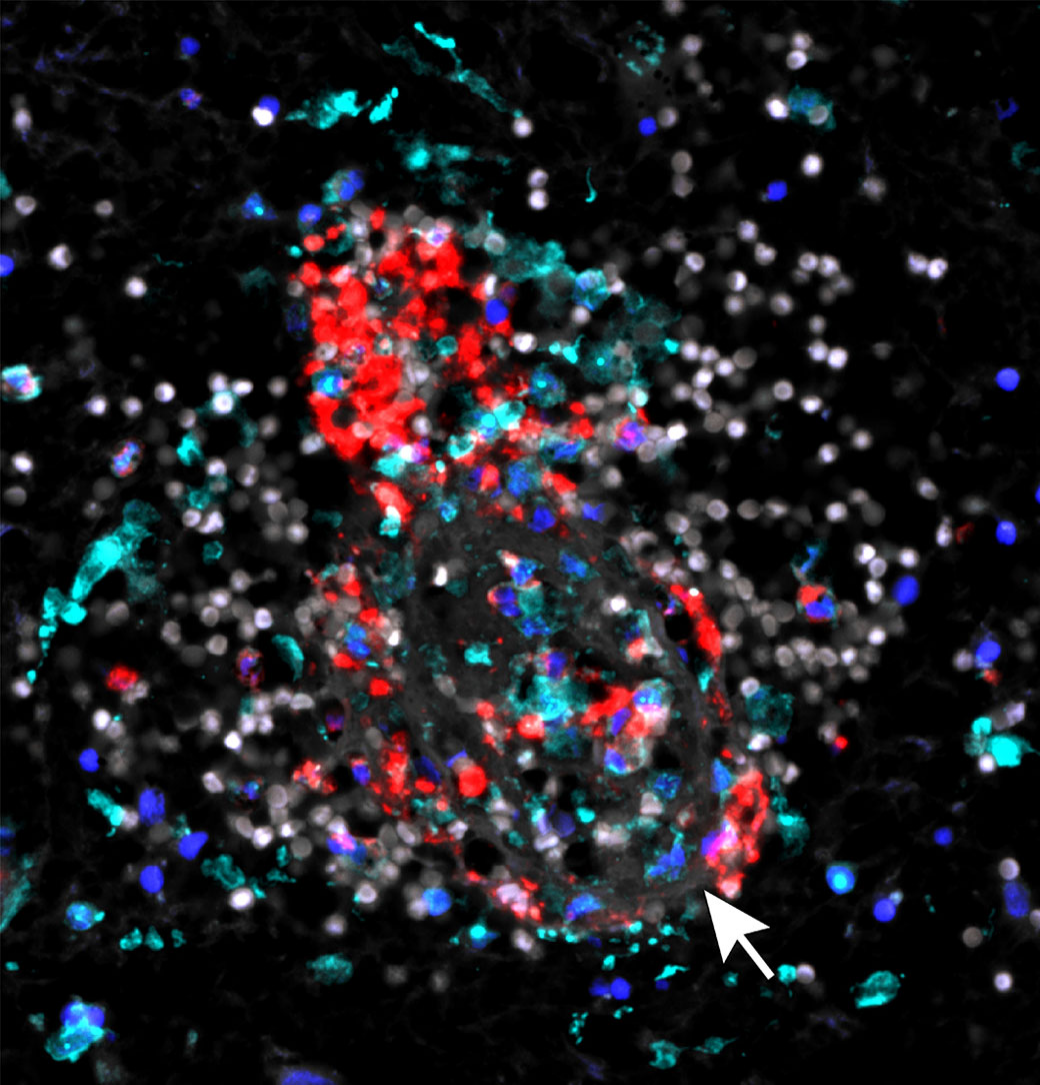
**The blood immune response in human intracerebral haemorrhage.** Iba1^+^ (cyan) microglia/macrophages in close proximity to CD11b^+^ (red) myeloid cells responding to red blood cells (white) around a diseased vessel (white arrow) in human brain tissue of an intracerebral haemorrhage case. DAPI is shown in blue. In our paper, we identify an important molecule that signals between these cells, impacting brain damage and resilience.

**Why did you choose DMM for your paper?**

I really enjoy reading papers published in DMM and our research fits the aims and scopes of the journal. On top of this, I have received travel award funding from DMM to present this work at an international conference and that also helped place DMM at the front of my mind!

We are in an era of data-rich science where papers are increasingly expansive and analysis of data is a job in itself

**Given your current role, what challenges do you face and what changes could improve the professional lives of other scientists in this role?**

If we could add an extra four hours to the day that would be perfect… If not, I think conversations need to be had about ever-increasing workloads of academic scientists. We are in an era of data-rich science where papers are increasingly expansive and analysis of data is a job in itself. On top of this, COVID forced communication/interactions online, and this has now become a mainstay of scientific interactions. I have certainly noticed a large uptick in the number of emails and online meetings since the world went back to a (new) normal post-COVID. All of these things steal time from our main jobs of studying the literature, advancing discovery, disseminating findings and training the next generations. I love my job, but I love science more, and I worry the latter will begin to suffer if we don't keep it centre focus of the former. If we want postdocs to produce ground-breaking science, we need to be surrounding them with wet- and dry-lab technical staff to reduce some of those tasks and open up that crucial time/space for creative thinking.

**What's next for you?**

I have a couple of projects ongoing at the moment. One continuing focus on ICH, but this time attempting to better understand the temporal trajectory of the immune response in human brain tissue. In the other project, I am using a mouse model of ischaemic stroke to understand the relationship between heterogeneous reactive microglial cell states.

**Tell us something interesting about yourself that wouldn't be on your CV**

I have a couple of interesting facts that normally surprise people; I broke my back in a sledging accident when I was 18, and I once represented Great Britain in the sport of pétanque.
